# Long-term stability of the MR system of the Philips Ingenuity TF

**DOI:** 10.1186/2197-7364-2-S1-A22

**Published:** 2015-05-18

**Authors:** Jarmo Teuho, Virva Saunavaara, Mika Teräs

**Affiliations:** Turku PET Center, Turku, Finland

The Philips Ingenuity TF is a sequential PET/MR with an Achieva 3T X-series MR and Gemini TF PET system connected with a rotating table. Both the PET and MR systems are designed to minimize mutual system interference. However, a longitudinal study concerning the stability of the MR system has not been conducted before. The stability of the MR system affects diffusion weighted imaging (DWI), diffusion tensor imaging (DTI) and functional MRI (fMRI), especially in longterm and multi-center studies. In addition, the variation of geometric distortions and consistency of MR image quality is important for clinical studies and radiotherapy planning. The long-term stability of the MR system was monitored by weekly quality control (QC) scans during one year. For measuring DWI, DTI and fMRI stability, protocols from fBIRN and vendor QA were implemented by using a spherical agar phantom and a liquid sphere phantom. For measuring the variations of image quality, ACR phantom measurements were performed. For the fBIRN and vendor QA protocols, automatic QC programs provided by the consortium and manufacturer were used. For the ACR phantom, an automatic quality control measurement was developed in MATLAB 2011b. Analysis of the QC scans did not show any significant variations with the fBIRN, vendor QA or ACR protocols. Thus, the MR system of the Ingenuity TF was deemed to be stable in the longitudinal study. Automated analysis of QC measurements was deemed necessary for implementing multiple, weekly measurements for PET/MR.

